# A Screening Approach for Classroom Acoustics Using Web-Based Listening Tests and Subjective Ratings

**DOI:** 10.1371/journal.pone.0116572

**Published:** 2015-01-23

**Authors:** Kerstin Persson Waye, Lennart Magnusson, Sofie Fredriksson, Ilona Croy

**Affiliations:** 1 Occupational and Environmental Medicine, The Sahlgrenska Academy at the University of Gothenburg, Gothenburg, Sweden; 2 Department for Clinical Neuroscience and Rehabilitation, Section for Audiology, University of Gothenburg, Gothenburg, Sweden; Kyoto University, JAPAN

## Abstract

**Background:**

Perception of speech is crucial in school where speech is the main mode of communication. The aim of the study was to evaluate whether a web based approach including listening tests and questionnaires could be used as a screening tool for poor classroom acoustics. The prime focus was the relation between pupils’ comprehension of speech, the classroom acoustics and their description of the acoustic qualities of the classroom.

**Methodology/Principal Findings:**

In total, 1106 pupils aged 13-19, from 59 classes and 38 schools in Sweden participated in a listening study using Hagerman’s sentences administered via Internet. Four listening conditions were applied: high and low background noise level and positions close and far away from the loudspeaker. The pupils described the acoustic quality of the classroom and teachers provided information on the physical features of the classroom using questionnaires.

**Conclusions/Significance:**

In 69% of the classes, at least three pupils described the sound environment as adverse and in 88% of the classes one or more pupil reported often having difficulties concentrating due to noise. The pupils’ comprehension of speech was strongly influenced by the background noise level (p<0.001) and distance to the loudspeakers (p<0.001). Of the physical classroom features, presence of suspended acoustic panels (p<0.05) and length of the classroom (p<0.01) predicted speech comprehension. Of the pupils’ descriptions of acoustic qualities, clattery significantly (p<0.05) predicted speech comprehension. Clattery was furthermore associated to difficulties understanding each other, while the description noisy was associated to concentration difficulties. The majority of classrooms do not seem to have an optimal sound environment. The pupil’s descriptions of acoustic qualities and listening tests can be one way of predicting sound conditions in the classroom.

## Introduction

Modern curriculum for elementary schools promotes teacher-based, individual-based as well as group-based teaching methods. These various forms of methods may be present at the same time and place, which puts high acoustic demands on the classroom. The room hence needs to support speaking and listening, both at close range and from a distance, and provide acceptable working conditions, also when several persons are talking at the same time as in group-based learning.

Basic room acoustics principles are well described elsewhere [1, 2], however when a room should support several acoustic functions, the optimal balance between reflective, absorbent and diffusive surfaces is quite a complex matter [3]. Speech is transmitted to the listeners via direct and reflected sounds, where direct sounds are most important for the listener close to the speaker and reflected sounds more important for distance listening. Speech perception is mainly degraded by a long reverberation time (the time it takes for a sound to decay 60 dB) and a high background sound level. An optimal reverberation time in classrooms is approximately around 0.5s for normal hearing children, while 0.3s may be required for children with hearing impairment [4, 5]. Problems with longer reverberation time may exist in older schools with acoustically hard surfaces, and surprisingly also in new schools with modern architecture and large surfaces of glass and stone material.

High background sound levels in schools are mainly a result of indoor sounds from activities and speech from the pupils themselves [6–8], and the typical background levels from ventilation and heating of 35–45dBA increase to about 55–70dBA when pupils enter the classroom [9–11]. The speech sounds and its relation to the background sound level is commonly referred to as signal to noise ratio (SNR), and in a learning situation, a SNR of +12 to +15dB is required [4, 12]. As a relaxed speaking teacher’s voice would be approximately 55dBA at 2 meters distance [12], in reality most of the time and especially at a distance from the teacher the optimal SNR is not met. This is particularly serious in a school situation as children in general, and non-native speaking children and children with hearing impairment in particular, are especially vulnerable to poor signal to noise ratios [13–15]. Previous research has accordingly established that poor listening conditions in classrooms impair speech comprehension, [16] and additionally also affect memory [17–19], increase annoyance and the mental effort needed to listen [20] and decrease school performance in general [21]. Furthermore, attention deficits may be enhanced in poor listening conditions as it has been hypothesized that chronic noise exposure results in loss of attention over time [22].

It can hence be concluded that the acoustic conditions in the classroom are of great importance for the pupils’ school achievement. In assessing listening conditions there are several indices based on physical room acoustic measures such as, speech clarity (C50) and speech transmission index (STI) [23, 24]. However, such assessments require measurements on site which are expensive and time consuming to carry out. Furthermore, we also need to improve our understanding of how objective measures relate to subjective perception of acoustic qualities and in different situations with regard to age and vulnerability [5, 25]. In large scale studies subjective measurements, including both speech perception testing and subjective ratings, may hence form an alternative way of screening for poor acoustic conditions. Subjective measures have previously been used to validate objective measures of speech intelligibility among normal hearing and hearing impaired listeners [26], while only a few studies have investigated how pupils perceive the acoustical quality in a typical classroom. In an extensive study comprising more than 2000 school children, the children rated sounds that they heard at school and sound that they perceived as annoying [20]. Relating measured sound levels to the subjective percepts, the authors concluded that children can be sensitive judges of their environment.

The present study was performed to evaluate whether a web based approach including listening tests and questionnaires could be used as a screening tool for poor classroom acoustics. We also aimed to assess how a large group of pupils experience their sound environment and how they felt it affected their school achievement. It was hypothesized that the teacher’s description of the classroom and its acoustic properties and the pupils’ description of sound the quality of their classroom would predict their speech perception. It was also hypothesized that poor room acoustics would be especially detrimental for the high background noise level condition and for the listening position far away from the speaker. The study lends support to the feasibility of a web based approach and identifies key perceptual elements of importance for listening conditions.

## Method

The study was part of the Researchers’ night, which is a program initiated by the European Commission and carried out in collaboration with researchers all over Europe, http://ec.europa.eu/research/researchersnight. The aim is primarily to promote interest for research among students and pupils. In Sweden the national research funds involved were: The Swedish Strategic Research (VR) Sweden’s Innovation Agency (VINNOVA) and Swedish Research Council for Health Working Life and Welfare (FORTE). The invitation to the schools, the instructions, the questionnaires and listening tests were all administered via internet, published on the Swedish home page: www.forskarfredag.se.

### Ethics Statement

The study followed the Declaration of Helsinki on Biomedical Research Involving Human Subjects.

### Participants

All classes at junior high school and grammar schools in Sweden were invited via the webpage. Those who expressed an interest to take part were included in the study. In total 59 classes from 38 schools in Sweden took part. In each class there were 7 to 42 pupils (mean 18.9, standard deviation 6.5), aged between 13 and 18 years (grammar school year 7–9; junior high school year 2–3). One class with 29 children was excluded from the analysis, because of from the group mean largely deviating results in the listening test. The average results of their listening test was 3.6%, where the next best class achieved 17.9%.

The total number of remaining pupils was 1106 with 49.4% girls and 51.6% boys. The characteristics of the classes are visualized in Table 1 Of the pupils, 179 (15.8%) did not have Swedish as their native language, 48 (4%) reported impaired hearing and 4 pupils had hearing aid.

**Table 1:** Characteristics of the participating classes.

**Number of classes and percentage**		**n**	**%**
**Characteristics of the pupils**			
Age	Class 7	16	27.6
	Class 8	24	41.4
	Class 9	11	19.0
	Gymnasium Class 2 or 3	7	12.1
Sex Distribution	Less than 40% girls	13	22.4
	40–60% girls	34	58.6
	More than 60% girls	11	19.0
Native language not Swedish	Less than 10% of the children	39	67.2
	10–50% of the children	11	19.0
	More than 50% of the children	7	12.1
	Not reported	1	1.7
Self-reported hearing problems	No child per class	25	43.1
	One child per class	17	29.3
	Two or three children per class	14	24.1
	Not reported	2	3.4
Hearing aids	No child per class	53	91.4
	One child per class	4	6.9
	Not reported	1	1.7
**Room acoustic features of the classroom**			
Number of hard walls	Less than 4	24	41.4
	All walls	34	58.6
Number of soft walls	No soft walls	39	67.2
	One or more soft walls	19	32.8
Number of shelves covering half of the wall	No Wall	27	46.6
	One wall	21	36.2
	Two or more walls	10	17.2
Acoustic panels	Don´t know	6	10.3
	Other absorbents	5	8.6
	Suspended acoustic panels	13	22.4
	Directly mounted acoustic panels	34	58.6
**Room volume**	**Range**	**Mean**	**SD**
Height	3–5m	3.0m	0.44m
Length	4–15m	8.9m	2.4m
Width	4–14m	7.5m	1.6m

The teacher responsible for each class was given a password and could then access the study information including the wave files with the listening tests and the questionnaires, and was also given the opportunity to submit the results via the web. The teacher was asked to give a description of the acoustic features of the classroom, including size of the room, the type of walls (acoustically hard or soft), the type of absorbing acoustic panels in the ceiling if any, and how they were mounted, (directly or suspended) and number of larger scattering objects, such as shelves. To guide the teachers, examples of acoustically soft (i.e. brick, perforated gypsum) and hard walls (windows, painted walls) were given. Furthermore, if they were not able to tell the acoustic properties of the ceiling, the alternative “do not know” was included, and this was reported by six teachers (10.3%). The characteristics of the participating classes are presented in Table 1.

### Listening tests

The pupils listened to standardized recordings with a female voice of Swedish sentences with low semantic redundancy [27]. The Hagerman test material consists of eleven lists each one comprised of ten different five-word sentences. The different sentences are constructed using a strict syntactical structure: proper noun, verb, numeral, adjective and noun. Each word in the sentences is randomly chosen out of ten possible words, for instance “Karin gave two old buttons”. For each listening condition, a list of 10 different sentences were presented and as every sentence had 5 words the pupils could have 0 to 50 (5*10) correct answers in each listening condition. The pupils gave their answers during each listening condition in a multiple choice task with 10 possible answers for each word. Each pupil gave the answer on his/her own sheet, the results were then summarized per class and calculated as percentage correct heard words per class for the different conditions and in total.

The test material was presented in four listening conditions: *low* and *high* SNR and *close* and *far away* from the loudspeakers. The noise signal was filtered to have the same long term spectrum as the speech material and was slightly (10%) amplitude modulated with a modulating noise that had most of its energy between 1 and 5 Hz [27, 28].

In the Low background condition, the SNR was-3dB, with the background noise presented at a level of 3dB above the speech. In the High background, the SNR was-6dB with the background noise presented at a level of 6dB above the speech. The SNRs were selected on the basis of a pilot study carried out in two classes at grammar schools age 16 years, not taking part in the study. The SNRs chosen corresponded to around 40 to 60% correct answers of the psychometric response function derived. The function shows the relationship between a test parameter (in this case SNR) and the corresponding subjective response for each SNR tested (in this case percentage correct answers).

The sentences were presented through loudspeakers connected to a computer or MP3 player, using the schools own playback system. The loudspeakers were placed at a position where the teacher usually stands when teaching the class. The volume was chosen before the test began to give a comfortable listening level determined by the pupils. Before the tests the pupils were given the opportunity to hear one practice list with ten sentences without noise. These sentences were not included in the final trial.

For the test, the class was divided into two groups, one positioned close to the loudspeakers (at the position of the first row of the classroom) and one far away from the loudspeaker (at the position of the back row of the classroom). After one listening test with low background noise level and one with high background noise level the groups changed places. Hence, each group heard the two signal-to-noise conditions twice, one in the close position and one in the far-away position, yielding four conditions in total. The whole procedure took about 45 minutes.

### Questionnaire

A questionnaire was distributed in addition to the listening test where the pupils were asked to give their description of the classroom acoustic qualities using the adjectives; quiet, clattering and noisy using the ratings: not at all, somewhat and very much. The questionnaire also included questions on disturbance of various noise sources such as other pupils talking, chair noise, noise from the corridor and traffic noise. The pupils were also asked to rate the interference of the sound environment with their ability to hear, talk and concentrate. The questionnaire is displayed in the S1 Table.

### Analyses

In order to protect children’s anonymity, the results from each class were reported as percentages of answers in the various categories, hence no individual analyses of relations etc. could be carried out.

Statistical analyses of the data were done using SPSS 19. The outcome of the listening tests with regard to signal to noise ratio and distance were analyzed using ANOVA for repeated measurements with the two factors distance and background sound level. As the initial analysis showed that no interaction effect was observed between the conditions, the data from the two distances and signal to noise ratios were combined in further analysis. As a first step also the influence of individual characteristics of the pupils (age, gender, native language, hearing impairment) were tested and as none contributed significantly (p>0.26), they were not considered in further analyses. The influence of the acoustic classroom features and subjective descriptions of the acoustic characteristics on the listening test were subsequently analyzed by linear regression with the combined listening data as dependent variable. In the analyses of physical acoustic features, the number of soft walls were highly correlated to the number of hard walls (r>0.6) and therefore both could not be included in the same model. The influence of the variables: width, length and height, ceiling absorbents (coded as dummy variables), number of shelves covering at least half the wall, and number of hard walls were added in the linear regression. Non-significant variables were removed by backward stepwise method; prob of entry = 0.05 and prob of removal = 0.10. Collinearity between explanatory variables was assessed using Pearson’s correlation or Spearman’s rank correlation, where applicable, and correlation below r = 0.6 was considered acceptable. The subjective descriptions of the sound environment (clattery, noisy, quiet) could not be deemed independent and were hence entered one by one in separate models.

The questionnaire data was analyzed descriptively in order to get an impression of how many children per class report a disturbing sound environment. The coherence between questionnaire variables or between variables and characteristics of the pupils were analyzed using Fishers exact test or Chi-Square test.

## Results

### Listening test—Perception of speech

**Figure 1:**
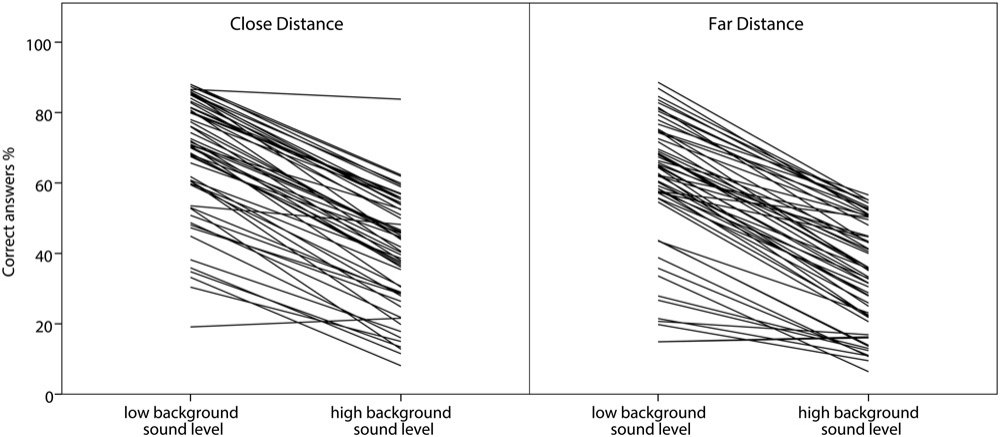
Percentages of the correct answers in the listening test. Each line represents one class. The diagrams display the difference between the high and low background sound levels divided into close and far distance. It can be seen that high background sound level worsened the results in 98% of the classes. The diagram also shows the effect of sitting close and far away to the loudspeakers. Far distance worsens the results in 93% of the classes.

The average percentages of correct answers for the four conditions of the listening test are given in Table 2. Increasing the background noise level by 3dB gave a highly significant reduction of correct answers in the listening test (F1,57 = 533.3; p<0.001). The classes performed on average 26.3 percentage points (pp) worse in the high compared to the low background noise level condition. Close distance on the other hand improved the results significantly by 6.8 pp (F1,58 = 58.3; p<0.001) and this was not affected by the signal to noise ratio (non-significant interaction effect). The results are visualized in Fig. 1.

**Table 2:** Mean percentage and standard deviation in brackets of correct answers in the listening test per class and conditions. A significant main effect of background sound level as well as of distance was revealed.

**Distance**	**Low background sound level (-3dB SNR)**	**High background sound level (-6dB SNR)**	**Combined background sound levels**
**Close**	67.4 (17.0)	40.6 (15.8)	*53.9 (15.9)*
**Far away**	59.9 (19.1)	34.2 (14.9)	*47.1 (16.4)*
***Combined distances***	*63.7 (17.7)*	*37.4 (15.0)*	
	*Difference between low and high background sound level = 26.3 percentage points*		*Difference between close and far distance = 6.8 percentage points*

There was a large variation between classes in percentage correct heard words, with a range of 18% to 72% in the averaged listening test conditions. However, 98% of the classes performed better with lower background noise level and 93% of the classes performed better in close compared to far distances, see Fig. 1.

### Factors that predicted the listening test

The linear regression of the factors related to the acoustic features and descriptors of the sound quality, hypothesised to predict the listening test, are given in Table 3. The variable suspended acoustic panels (p = 0.02), and length of the room (p = 0.006) were found to significantly predict correct answers on the listening test, while number of shelves missed significance. The model excluded acoustic panels directly mounted on the ceiling, number of hard walls, classroom width and height as non-significant contributors. It should be noted that there was a significant association of presence of acoustic panels (suspended or directly mounted) and number of hard walls (p = 0.005 Fishers exact test) indicating that classrooms with higher number of hard walls also more often had acoustic panels, however the hard walls did not contribute significantly to the model. Of the subjective descriptions of the sound environment (clattery, noisy, quiet), only clattery was found to significantly predict the outcome of the listening test. The negative association indicates that the greater number of pupils who describe the sound environment as very clattery, the lower was the correct scores on the listening test.

**Table 3:** Point estimates of effect (unstandardized coefficient, B) and standard error (SE), 95% confidence intervals of B (95% CI) from linear regression models for the total combined answers over distances and signal to noise ratios (dependent variable).

**Category Explanatory variables**	**B (SE)**	**(95% CI)**	**p-value**
**Physical features ^¤^**			
Acoustic panels^#^ suspended	10.93 (4.58)	(1.73–20.14)	0.02
Length m	2.29 (0.74)	(0.70–3.88)	0.006
No of shelves^#^	-4.36 (2.57)	(-9.52–0.78)	0.09
**Subjective descriptions** ^¥^			
Clattery	-0.46 (0.22)	(-0.90–-0.25)	0.038

The physical parameters in each category were entered and non-significant variables removed by backward stepwise method; prob of entry = 0.05 and prob of removal = 0.10. Subjective descriptions were not considered to be independent and were hence entered one by one in the model.

^¤^ width, height, number of hard walls, excluded in the model,

^#^ acoustic panels directly mounted and suspended from the ceiling added as dummy variables with “other” as reference category, directly mounted excluded in the model.

number of shelves added as: zero, one or more than one.

^¥^ loud, not quiet excluded in the model

**Figure 2:**
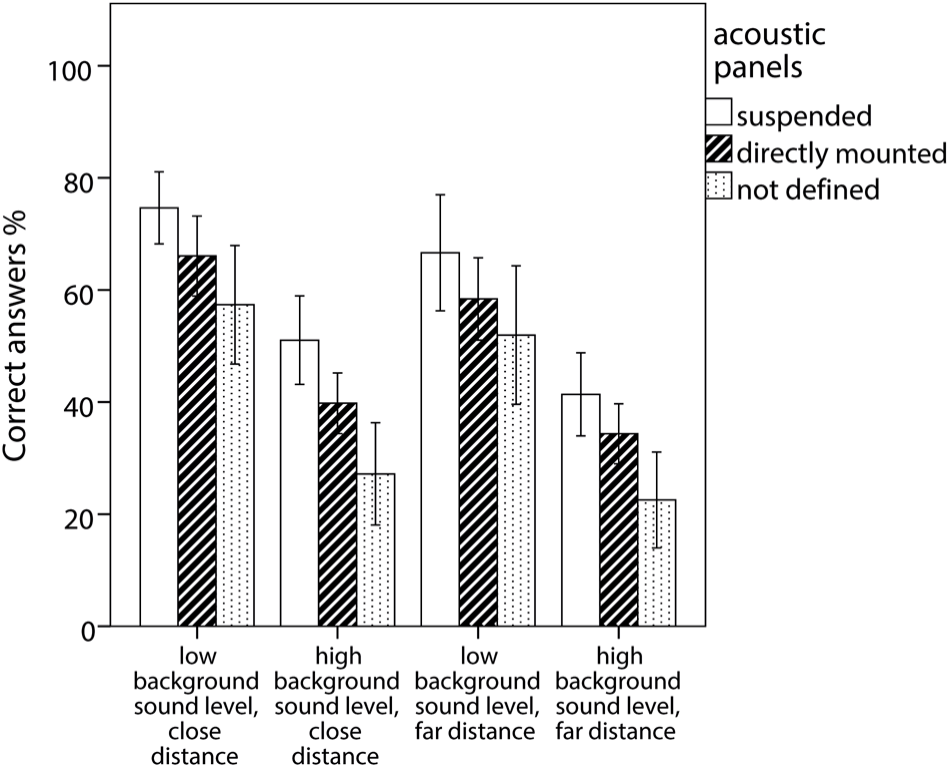
Average percentage of correct answers and 95% CI for classes divided into those with suspended or directly mounted absorbing panels or classes where the teacher did not know about absorbing panels.

**Figure 3:**
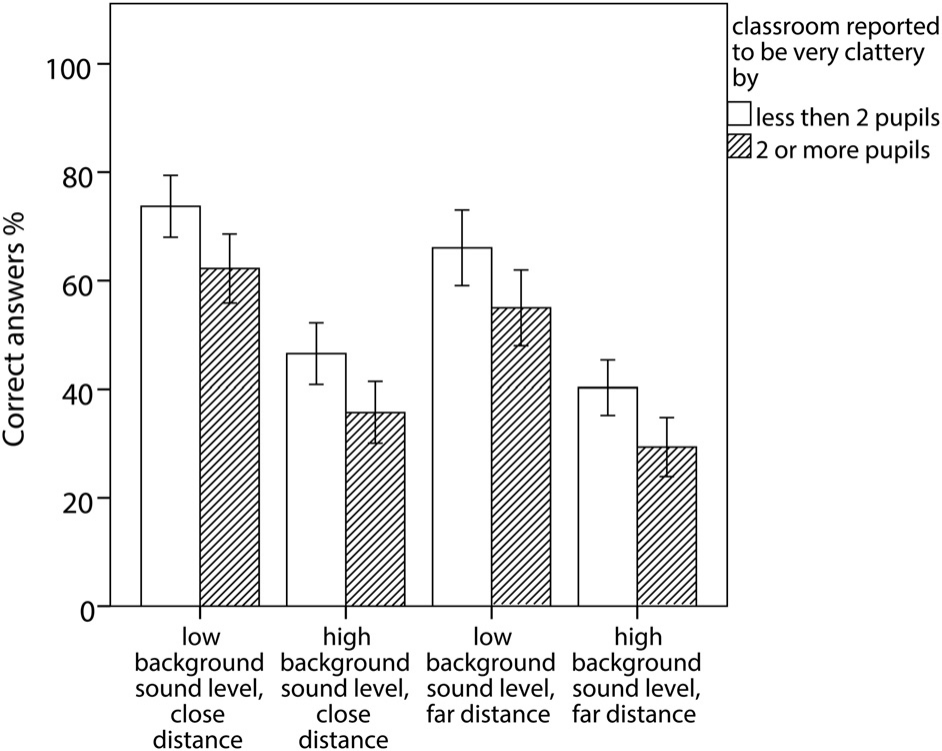
Average percentage of correct answers and 95% CI for classes divided into those with two or more respective less than two pupils describing the classroom as very clattery.

The two most important predictive factors are illustrated for the four listening conditions in Fig. 2 and Fig. 3.

### Results of the questionnaire

**Disturbing sound environment in the classroom.** In all classes except one (98% of the classes), at least one child described the classroom as not quiet, as very clattery or as very noisy. In 69% of the classes three or more children described the classroom as not quiet, as very clattery or as very noisy (compare Table 4). Acoustic panels were found to have a positive effect on the perceived clattery, as classrooms with any acoustic panels were less often judged as being very clattery (Chi Square = 7.9, p = 0.046).

**Table 4:** Results of the questionnaire: Interference of the sound environment with pupils’ ability to communicate, listen and concentrate and descriptions of the sound quality aspects of the classroom.

**Number of children per class who affirm to the statement**								
	**no child**		**one child**		**two children**		**three or more**	
	n	%	n	%	n	%	n	%
**Description of sound qualities**								
The classroom is …								
… not quiet	8	13.8	13	22.4	5	8.6	32	55.2
… very clattery	10	17.2	16	27.6	14	24.1	18	31.0
… very noisy	13	22.4	15	25.9	10	17.2	20	34.5
Combined	1	1.7	8	13.8	9	15.5	40	69.0
**Source of disturbance**								
I am often disturbed by…								
… others talking	5	8.6	11	19.0	9	15.5	33	56.9
… chair noise	20	34.5	15	25.9	9	15.5	14	24.1
… noise from the corridor	22	37.9	13	22.4	11	19.0	11	19.0
… traffic noise	33	56.9	13	22.4	7	12.1	3	5.2
**Interference of sound environment with ability to talk, listen and concentrate**								
I find it often hard to …								
… understand other pupils	28	48.3	15	25.9	8	13.8	4	6.9
… understand the teacher	35	60.3	12	20.7	7	12.1	2	3.4
… get heard	24	41.4	16	27.6	11	19.0	6	10.3
… concentrate	7	12.1	12	20.7	15	25.9	24	41.4

There was no significant coherence between the age of the pupils, native language or the sex distribution with the description of the sound quality of the classroom.

The most disturbing sound source was other pupils. In more than half of the classes, three or more pupils reported disturbance from others talking. Disturbance by scraping noise from chairs and noise from the corridor were reported by three or more children in 24% and 19%. Traffic noise was considered to be disturbing by only a few classes.

**Interference of sound environment with the ability to talk, listen and concentrate.** In 40% of the classes there was at least one pupil that often had difficulties hearing what the teacher said, compare Table 4. Furthermore, in 88% of the classes one or more pupil often reported difficulties concentrating due to noise. There was a significant coherence between the rating of the sound environment in the classroom as noisy and the rating of concentration abilities (Chi Square = 20.8, p = 0.013), indicating that in those classrooms which were perceived as very noisy, more children reported concentration difficulties. Similarly, the rating of the sound environment as clattery was associated with the number of pupils reporting it difficult to understand each other (Chi-Square = 11.9, p = 0.008). There was also a significant coherence between presence of any acoustic panels and number of children reporting it hard to understand the teacher (Chi Square = 9.3, p = 0.02). No significant coherence between the age of the pupils, native language or the sex distribution was found with the interference of sound.

## Discussion

To be able to perceive speech is crucial for school performance where speech is the main mode of communication and teaching. However, the vast majority of classrooms do not seem to have an optimal sound environment that supports speech transmission and speech comprehension. In 86% of the classes in this study one or more of the pupils described adverse sound conditions in the classroom and in 88% of the classes one or more pupils described often having problems concentrating because of noise. The inability to concentrate was related to how noisy the classroom was perceived. The pupils described the classrooms sound condition as less adverse if there were acoustic panels in the classroom and reported it to be less hard to understand the teacher if acoustic panels were present. The problem can not be neglected as there was at least one pupil in about 40% of the classes who often reported problems in hearing what the teacher said. The reporting of the classroom as clattery was also related to problems understanding of what other pupils said, a problem reported by one or more pupils in 52% of the classes. However, measured room acoustics and/ or pupils description may not fully describe the actual communication situation and therefore the results of the listening test are of importance.

Here, we found pupils speech perception to be worse in the higher background noise levels, which was to be expected based on previous research. This has previously been shown in a classical study where both normal hearing and hearing impaired children showed lower speech perception for monosyllabic words with decreasing SNR, as well as in conditions with longer reverberation time [14]. In that study the least favourable SNR used was 0 dB while we used-3 and-6 dB SNR. It can be noted that the normal hearing children in their study decreased their speech perception by 23.6 percentage points or from 71.3% to 47.7% when decreasing the SNR from +6 to 0 dB, in a condition with 0.4 seconds reverberation time. As we used sentence material and they used monosyllabic words, the results are not directly comparable, but it is interesting to see the similarity of the results with our study giving an average decrease in performance by 26.3 percentage points when the SNR was reduced from -6 to-3dB SNR in the combined distance evaluation. [14] also found that the detrimental effect of a combination of a low SNR and long reverberation time was greater than the sum of the individual factors per se, further emphasising the importance of a good acoustic support of the classroom. Importantly, the pupils’ perception of the classroom being clattery significantly predicted the results of the listening test. This indicates that the pupils’ perception of the sound environment may be used as one predictor for good acoustic conditions.

We could not find that age, sex distribution, hearing loss and/or native language had a significant influence on the results of the speech perception test. This is in contrast to what has previously been described in the literature that young children, non-native listeners and/or hearing impaired individuals have greater difficulty perceiving speech in less favourable listening conditions [29, 30]. The lack of significant finding was most likely due to the limited range in age (13–18 years) between classes and/or a result of the data acquirement with summaries on class level, giving little possibility to perform more individual orientated analyses.

Sitting close to the loudspeakers helped the pupils to perceive the sentences better in both background noise conditions, implying that a position close to the teacher is of advantage for the pupils. As obviously not all of the children can sit close to the teacher, classroom acoustics need to support listening conditions also at a distance. Here, of all the variables we recorded, only presence of suspended acoustic panels and more unexpected length of the classroom had a positive effect on speech comprehension. To reduce reverberation time acoustic panels are usually placed on the ceiling and on the walls. The choice and placement are of importance though. Acoustic panels placed suspended from the surface generally have an improved absorbance of the lower frequencies, hence providing a more uniform absorbance across the frequency range, which improves speech intelligibility as compared to panels mounted directly onto the surface [31, 32]. Accordingly, the statistical analyses and as displayed in Fig. 2 indicate a stronger positive effect on speech comprehension of suspended acoustic panels as opposed to directly mounted, however this should be further investigated in studies with a larger number of classrooms with different acoustic features.

It is less obvious why length would predict speech comprehension. Length was correlated to the presence of acoustic panels, which may explain part of the effect. Other reasons are purely speculative, but it is possible that the volume of the playback system was increased for a longer classroom and that this per se made it somehow easier to score better on the listening test. This will need to be investigated in further studies using a more controlled set-up.

We are aware that there are some limitations in the study. The study was carried out via internet, with limited possibilities to control for errors that may occur in performing the study. However, only one of the classes had to be excluded because the results of their listening test were three standard deviations below the others and very likely caused by technical problems with setting up the experiment. 98% of the other classes performed as expected better in low than in high background sound level, which makes us optimistic that there were no major errors in the realization of the experiment. The way of distribution also means that we have little knowledge on the participating schools and those who chose not to participate. Furthermore, we have little control of the school’s playback system and how the quality influenced the results. The conclusions are also hampered by the fact that we only got summaries of the results per class, making it impossible to for instance relate individual reactions and perceptions to the results of the listening test. Although the listening test used seem to have performed well enough to detect room acoustic influences, future studies should address if test developments could improve the possibility to discriminate between individual characteristics.

Nevertheless, the results from more than 1000 pupils suggest that classroom sound environment is not optimal for the majority of classes. Acoustic panels in the classroom improve pupil’s rating of the classroom sound conditions and speech perception. The ability to understand the teacher and other pupils is vital for good school performance. It is therefore of high importance to enhance acoustic conditions and in doing so School administrates may rely on pupil’s perception and listening tests as one factor describing the sound conditions in the classroom.

## Supporting Information

S1 Table Questionnaire answered by the pupils. (DOCX)
