# Supplementary material for: A Screening Approach for Classroom Acoustics Using Web-Based Listening Tests and Subjective Ratings
Source: PLoS One. 2015 Jan 23;10(1):e0116572. doi: 10.1371/journal.pone.0116572 (PMC4304827; doi:10.1371/journal.pone.0116572)
Supplement: S1 Table — (DOCX) [file pone.0116572.s001.docx]

Table S1.

| **How do you experience the classroom in terms of acoustics and sound?** | | | | | | | |
| --- | --- | --- | --- | --- | --- | --- | --- |
| I find it hard to hear what other pupils say |  |  | seldom |  | sometimes |  | often |
| I find it hard to hear what the teacher says |  |  | seldom |  | sometimes |  | often |
| I find it hard to get myself heard |  |  | seldom |  | sometimes |  | often |
| I find it hard to concentrate because of noise |  |  | seldom |  | sometimes |  | often |
| I am disturbed by other pupils talking |  |  | seldom |  | sometimes |  | often |
| I am disturbed by chair noise |  |  | seldom |  | sometimes |  | often |
| I am disturbed by noise from the corridor |  |  | seldom |  | sometimes |  | often |
| I am disturbed by traffic noise |  |  | seldom |  | sometimes |  | often |
| **Please indicate how the following descriptions of the sound environment in the classroom correspond with your opinion** | | | | | | | |
| Quiet |  |  | very much |  | somewhat |  | not at all |
| Clattery |  |  | very much |  | somewhat |  | not at all |
| Noisy |  |  | very much |  | somewhat |  | not at all |
| **Some questions about you** | | | | | | | |
| Do you have hearing loss? |  |  | yes |  | no |  | I don´t know |
| Do you use hearing aids? |  |  | yes |  | no |  |  |
| Is Swedish your native language? |  |  | yes |  | no |  |  |
